# A Novel Paper-Based Electrochemical Biosensor Based on N,O-Rich Covalent Organic Frameworks for Carbaryl Detection

**DOI:** 10.3390/bios12100899

**Published:** 2022-10-20

**Authors:** Yawen Xiao, Na Wu, Li Wang, Lili Chen

**Affiliations:** National Engineering Research Center for Carbohydrate Synthesis, Key Lab of Fluorine and Silicon for Energy Materials and Chemistry of Ministry of Education, College of Chemistry and Chemical Engineering, Jiangxi Normal University, Nanchang 330022, China

**Keywords:** acetylcholinesterase, covalent organic framework, carbaryl, electrochemical biosensor [Fe(CN)_6_]^3−/4−^, paper-based electrode

## Abstract

A new N,O-rich covalent organic framework (COF_DHNDA-BTH_) was synthesized by an amine-aldehyde condensation reaction between 2,6-dialdehyde-1,5-dihydroxynaphthalene (DHNDA) and 1,3,5-phenyltriformylhydrazine (BTH) for carbaryl detection. The free NH, OH, and C=O groups of COF_DHNDA-BTH_ not only covalently couples with acetylcholinesterase (AChE) into the pores of COF_DHNDA-BTH_, but also greatly improves the catalytic activity of AChE in the constrained environment of COF_DHNDA-BTH_’s pore. Under the catalysis of AChE, the acetylthiocholine (ATCl) was decomposed into positively charged thiocholine (TCl), which was captured on the COF_DHNDA-BTH_ modified electrode. The positive charges of TCl can attract anionic probe [Fe(CN)_6_]^3−/4−^ on the COF_DHNDA-BTH_-modified electrode to show a good oxidation peak at 0.25 V (versus a saturated calomel electrode). The carbaryl detection can inhibit the activity of AChE, resulting in the decrease in the oxidation peak. Therefore, a turn-off electrochemical carbaryl biosensor based on a flexible carbon paper electrode loaded with COF_DHNDA-BTH_ and AChE was constructed using the oxidation peak of an anionic probe [Fe(CN)_6_]^3−/4−^ as the detection signal. The detection limit was 0.16 μM (S/N = 3), and the linear range was 0.48~35.0 μM. The sensor has good selectivity, repeatability, and stability, and has a good application prospect in pesticide detection.

## 1. Introduction

Pesticide residues and their degradation products are the main pollutants that pose great potential risks to the ecosystem and human health. They can destroy the ecological balance in an ecosystem and the main systems of the human body, such as the immune, nerve, and endocrine systems, and cause various diseases [1,2,3,4]. Carbaryl is one of the most common carbamate insecticides, named 1-naphthalene carbamate. Carbaryl enters the ecological food chain and endangers human health due to the biological accumulation and soil accumulation of carbaryl [5,6,7,8]. Moreover, carbaryl interferes with the hydrolysis of neurotransmitter acetylcholine (ATCl) and affects the normal secretion of the neurotransmitter. Therefore, it is very important to rapidly, reliably, and quantitatively detect carbaryl. At present, the commonly used methods of detecting carbaryl include chromatography [9], spectrophotometry [10], and immunoassay [11]. These methods are sensitive and reliable. However, their instruments are expensive, the sample pretreatments are cumbersome and time-consuming, and they require a trained person for operation and complex sample pretreatment. Electrochemical sensors are rapid, simple, sensitive, reliable, and low cost, which make up for the shortcomings of the above technologies and meet the requirements of portable detection [12,13,14,15,16,17,18,19,20]. Electrochemical sensors have broad development space and application prospects in the rapid detection of pesticide residues [21,22].

At present, electrochemical biosensors based on acetylcholinesterase (AChE) have attracted extensive attention in the detection of carbamate pesticides such as carbaryl because of their advantages in terms of nontoxicity, simplicity, high specificity, and high sensitivity [23,24]. For example, Lara et al. constructed an electrochemical biosensor based on a polypyrrole nanocomposite loaded with AChE to detect carbaryl [25]. The detection mechanism is carbamate pesticides binding to AChE [26,27], resulting in the esterification of the hydroxyl groups of the serine residues at the enzyme active site by carbamate to inactivate AChE. Afterward, the production of thiocholine (TCl), the electroactive product produced by its catalytic hydrolysis of ATCl, is reduced and the response current is reduced to achieve the detection of pesticides [28,29,30]. Yang et al. reported the performance analysis of a biosensor based on AChE. The bioactivity of the immobilized AChE and the direct electron transfer rate between the enzyme and the electrode affect the performance of the biosensor. These analyses provided guidance for the development of a new AChE-based biosensor for detecting organophosphorus pesticides [31]. Despite the AChE electrochemical biosensors having bright prospects, some concerns cannot be ignored. For example, it is challenging to maintain or improve the catalytic activity of AChE fixed to an electrode surface. In addition, the oxidation overpotential of TCl is reduced by the catalytic reduction of nanomaterial. For example, AChE/Prussian blue (PB)–chitosan (CHIT) showed good catalytic performance, which greatly reduced the oxidation potential of TCl generated by hydrolysis from 0.68 to 0.32 V [32,33,34]. However, the oxidation potential of 0.32 V still causes significant interference. Therefore, it is necessary to develop a portable electrode and suitable support material to fix the enzyme and to reduce the detection potential for portable and selective detection [35,36,37]. 

Covalent organic frameworks (COFs) are organic porous crystalline materials constructed by self-assembly of various building motifs [38,39]. The COFs have good biocompatibility and chemical stability, high specific surface area, and high porosity, and are easy to modify and functionalize. The highly ordered π–π conjugate system and independent open regular channels are very conducive to electron and mass transfer [40]. Due to the variety of methods for synthesizing COFs and the variety of monomers with different functional groups, their structures and properties are also abundant and varied, which result in the extensive application of COFs in various fields [41,42,43,44,45,46]. The abundant functional groups, good biocompatibility, and adjustable hole make the COFs particularly suitable for attaching biomolecules to build biosensors, which have been proven by some successful works [47,48,49]. 

In this work, 1,3,5-phenyltrimethylhydrazine (BTH) and 2,6-diuronic-1,5-dihydroxynaphthalene (DHNDA) were used to synthesize COF_DHNDA-BTH_ rich in N and O through the amine–aldehyde condensation reaction. Then, an electrochemical biosensor for the detection of carbaryl was prepared by loading AChE. The free NH, OH, and C=O groups on COF_DHNDA-BTH_ can covalently couple with AChE. Moreover, it has excellent biocompatibility. During detection, it can also interact with TCl through hydrogen bonding, so as to better capture the detection signal. The biosensor has a low detection limit and wide linear range. This method has a low detection potential (0.25 V versus saturated calomel electrode) and good reproducibility, stability and anti-interference ability. This good analytical performance proves that this strategy paves a new way for the design of portable AChE biosensors.

## 2. Experimental Materials and Methods

### 2.1. Materials and Reagents

BTH and DHNDA were purchased from Jilin Scientific Research Technology, Co., Ltd. (Changchun, China). N,N-dimethylacetamide (DMF), trimethylbenzene, acetone, dichloromethane, anhydrous ethanol, acetic acid (AcOH), AChE, ATCl, K_3_Fe[(CN)_6_]^3−/4−^, Na_2_HPO_4_, NaH_2_PO_4_, ascorbic acid (AA), glucose, KCl, NaNO_3_, CuCl_2_, HgSO_4_, CdCl_2_, and PbCl_2_ were purchased from Inokai Co., Ltd. (Beijing, China). Carbaryl was purchased from Jiangxi Mackenech Technology Co., Ltd. (Nanchang, China). All solutions are prepared using ultra-pure water purified by a Millipore-Q purification system (18.2 MΩ cm^−1^). Flexible carbon papers were purchased from Beichuan Co., Ltd. (Shanghai, China). White nail polish was purchased from Clio Co., Ltd. (Guangzhou, China). 

### 2.2. Instruments

Transmission electron microscope (TEM) images were obtained with a JEM-2010 (HR) (Japan Electronics Co., Ltd., Tokyo, Japan). Scanning electron microscope (SEM) images were obtained with a HITACHI S-3400N SEM at an acceleration voltage of 15 KV (Hitachi Limited, Tokyo, Japan). Fourier transform infrared spectroscopy (FTIR) analysis was performed using a Perkin-Elmer Spectro100 from Perkin-Elmer Corporation (Perkin-Elmer, Waltham, MA, USA). Brunauer–Emmett–Teller (BET) surface areas and pore size distributions were measured using N_2_ adsorption/desorption isotherms, which were tested with an Autosorb-iQ under 77 K (Quantachrome, Boynton Beach, FL, USA). X-ray diffraction (XRD) analysis was performed on a BRUCKER D8 ADVANCE X-ray powder diffractometer with scanning steps of 3°/min in a scanning range of 0–50° (Rigaku Corporation, Tokyo, Japan). All electrochemical characterization was performed with a CHI 760D (Shanghai, China).

### 2.3. Preparation of COF_DHNDA-BTH_

**Scheme 1:**
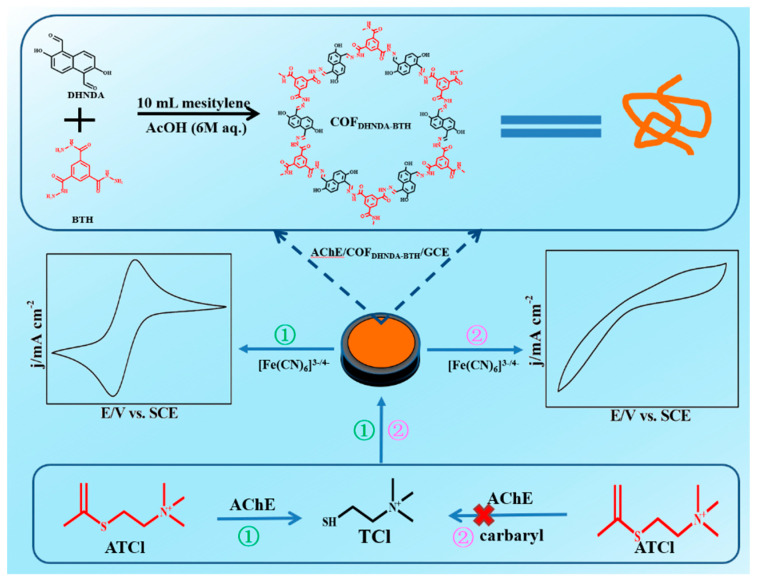
Schematic diagram of preparation process of COF_DHNDA-BTH_ and detection principle of electrochemical sensor based on AChE/COF_DHNDA-BTH_/GCE.

We dissolved 50 mg (0.2 mM) of BTH and 42 mg (0.3 mM) of DHNDA in 5 mL of mesitylene by ultrasound. Then, the solution was transferred to a reaction kettle with 3 mL of 6 M AcOH, and the solution was heated to 120 °C for 72 h. The obtained solid powder was washed with DMF, acetone, methylene chloride, and anhydrous ethanol, separately. Finally, the powder was dried in the oven at 60 °C to obtain COF_DHNDA-BTH_. The prepared procedures are shown in Scheme 1.

### 2.4. Preparation of AChE/COF_DHNDA-BTH_/Glassy Carbon Electrode (GCE)

Firstly, the surface of GCE was polished with Al_2_O_3_ powder and washed with ethanol and ultra-pure water until the surface of GCE was smooth and clean. Then, 2 mg was dispersed into 1 mL of ultra-pure water to obtain 2 mg/mL of aqueous suspension. Next, 10 μL of 2 mg/mL COF_DHNDA-BTH_ was dropped on the polished GCE surface. After 5 h, the COF_DHNDA-BTH_/GCE was rinsed with ultra-pure water. After that, 4 μL of 0.2 mg/mL AChE was dropped on the surface of the COF_DHNDA-BTH_/GCE. It was stored in a refrigerator at 4 °C. Finally, the electrode was rinsed with ultra-pure water to obtain AChE/COF_DHNDA-BTH_/GCE. The subsequent detection mechanism is shown in Scheme 1.

### 2.5. Preparation of Paper-Based Electrode 

**Scheme 2:**
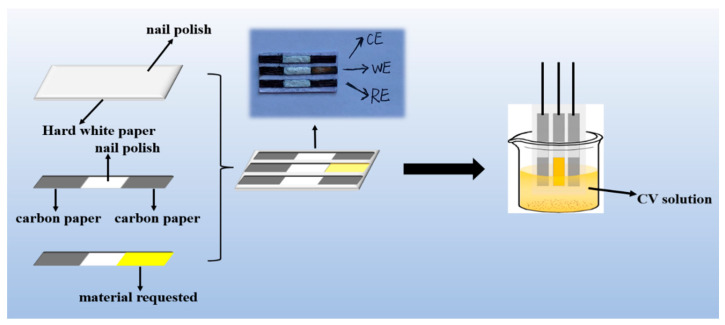
Preparation process of paper-based electrode and the picture of obtained paper-based electrode.

To prepare a portable AChE biosensor, a portable paper-based electrode was constructed as follows: First, the commercial carbon paper was cut into three long strips 3 mm in width and 3 cm in length. Next, both sides of a piece of white cardboard, 2 cm in width and 3 cm in length, were painted with white nail polish. Next, the three long carbon paper strips were pasted onto one side of the white cardboard in intervals of about 0.5 cm. The middle sections of the long carbon paper strips (about one-third of the long strips) were painted with white nail polish. The bottom section of long carbon paper strips was peeled off with acrylic transparent tape to obtain a new surface with graphene-like foam as a working electrode (WE), reference electrode (RE), and counter electrode (CE). The active areas of WE, RE, and CE were all 1 × 3 mm. The prepared procedures and a picture of the obtained paper-based electrode are shown in Scheme 2.

### 2.6. Preparation of AChE/COF_DHNDA-BTH_/Portable Paper-Based Electrode

The preparation procedures of the AChE/COF_DHNDA-BTH_/portable paper-based electrode were similar to that of AChE/COF_DHNDA-BTH_/GCE. Briefly, 10 μL of 2 mg/mL COF_DHNDA-BTH_ was dropped on the surface of WE on portable paper-based electrode. Then, 4 μL of 0.2 mg/mL AChE was dropped on the COF_DHNDA-BTH_/portable paper-based electrode to obtain an AChE/COF_DHNDA-BTH_/portable paper-based electrode. 

## 3. Results and Discussion

### 3.1. Characterization of COF_DHNDA-BTH_

**Figure 1:**
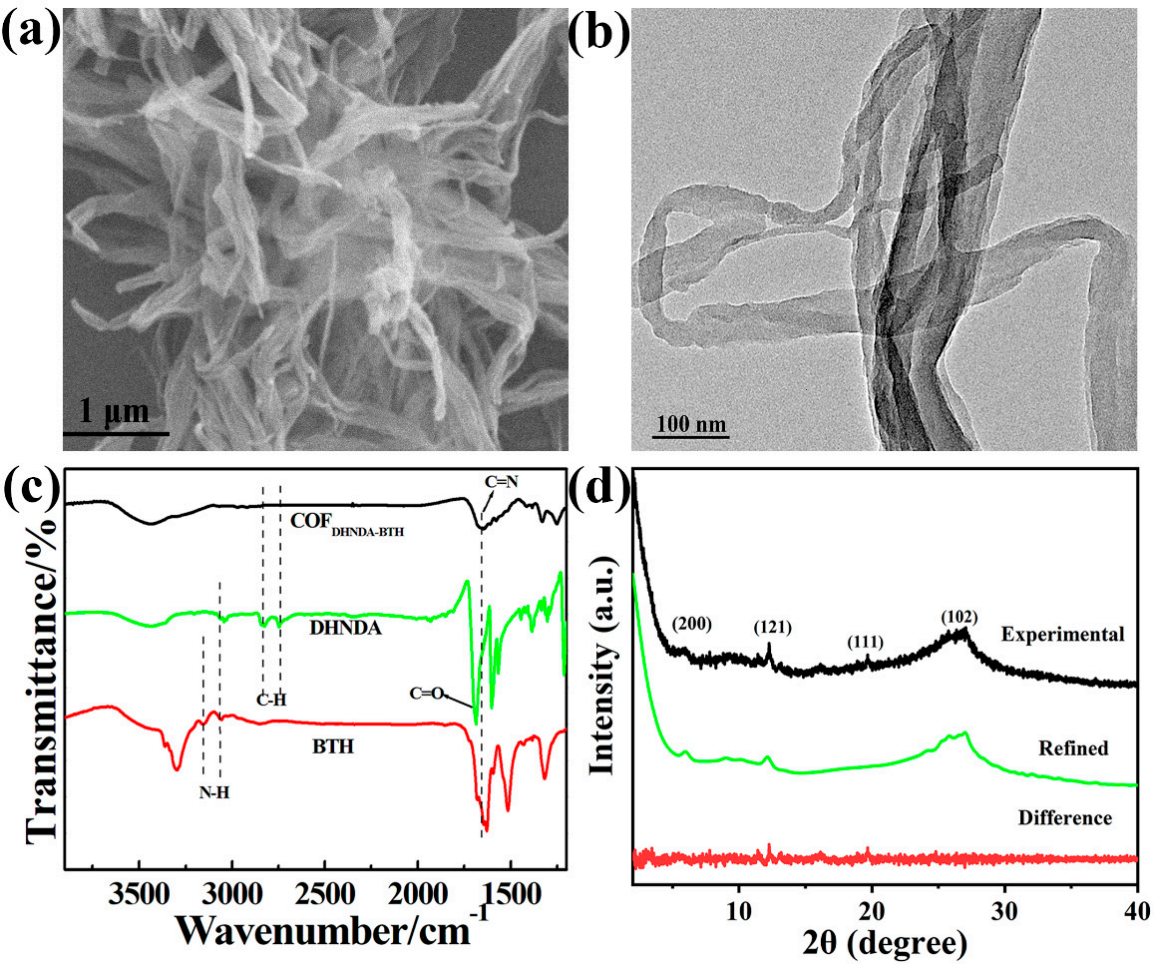
(**a**) SEM image of COF_DHNDA-BTH_. (**b**) TEM image of COF_DHNDA-BTH_. (**c**) FTIR spectra of DHNDA, BTH, and COF_DHNDA-BTH_. (**d**) Experimental and refined XRD pattern as well as the difference.

The SEM (Figure 1a) and TEM (Figure 1b) images showed that the as-prepared COF_DHNDA-BTH_ presented a nanoflower-like morphology that was composited of many two-dimensional (2D) nanoribbons with good flexibility based on π–π stacking. As shown in Scheme 1, these nanoribbons were formed by the amine–aldehyde condensation reaction between BTH and DHNDA. With good flexibility, the thickness of nearly a single molecule, as well as free NH, OH, and C=O groups, the COF_DHNDA-BTH_ can better load enzymes and assemble on the electrode surface, which is good for constructing biosensors based on COF_DHNDA-BTH_. The FTIR spectrum (Figure 1c) showed the O=C-H peaks of DHNDA at 2744 and 2836 cm^−1^, the C=O peak at 1692 cm^−1^ (green curve), and N-H peaks of BTH at 3058 and 3156 cm^−1^ (red curve). In the FTIR spectrum of COF_DHNDA-BTH_ (black curve), a peak of -C=N- appeared at 1641 cm^−1^, indicating the successful occurrence of an amine–aldehyde condensation reaction, which further proved the successful synthesis of COF_DHNDA-BTH_. The XRD patterns of COF_DHNDA-BTH_ showed many peaks at 5.95°, 12.27°, 19.67°, and 27.06°, corresponding to (200), (121), (111), and (102) crystal planes, respectively, indicating that the as-prepared COF_DHNDA-BTH_ had good crystallinity (Figure 1d). The AA and AB model obtained by simulation were compared with the experimental result, in which the XRD pattern agreed with the simulated AA stacking pattern (Figure S1, Supporting Information) and showed the layered structure with a layer distance of 3.58 Å (Figures S2 and S3, Supporting Information). The N_2_ adsorption/desorption isotherm of COF_DHNDA-BTH_ showed that the BET specific surface area was 46.91 m^2^ g^−1^ (Figure S4, Supporting Information). The aperture distribution of COF_DHNDA-BTH_ showed that the aperture was 1.379 nm (Figure S5, Supporting Information).

### 3.2. Characterization of Commercial Carbon Paper 

**Figure 2:**
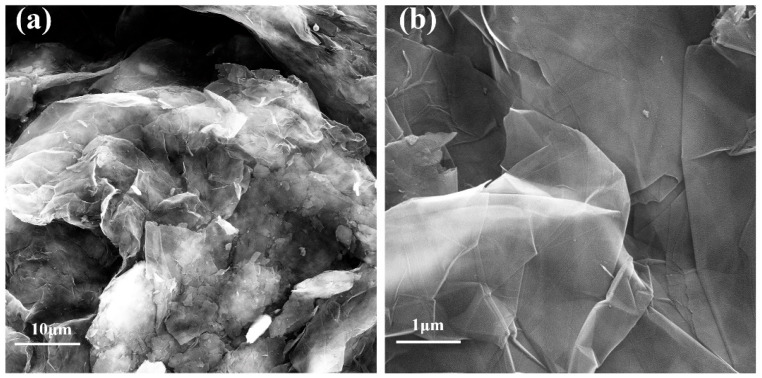
(**a**) Low-resolution and (**b**) high-resolution SEM image of commercial carbon paper.

Figure 2a,b show the morphology and structure of commercial carbon paper with high and low magnification, respectively. The figures reveal a rough surface with graphene-like foam, which was more conducive to loading active materials and to transferring electrons and electrolyte ions. 

### 3.3. Electrochemical Behaviors of AChE/COF_DHNDA-BTH_/GCE

**Figure 3:**
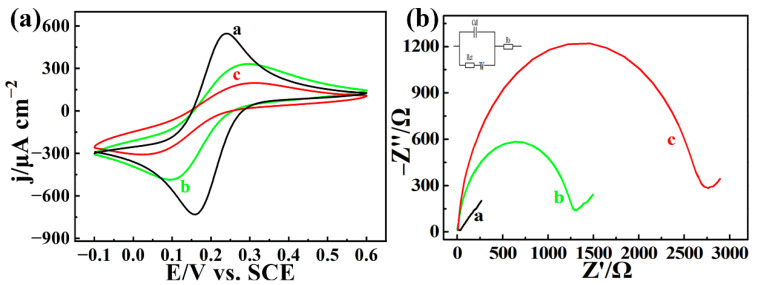
(**a**) CVs and (**b**) EIS of GCE (curve a), COF_DHNDA-BTH_/GCE (curve b), and AChE/COF_DHNDA-BTH_/GCE (curve c) in 0.1 M KCl with 5.0 mM [Fe(CN)_6_]^3^^−/4−^.

Figure 3 shows the CVs (Figure 3a) and EIS curves (Figure 3b) of GCE (curve a), COF_DHNDA-BTH_/GCE (curve b) and AChE/COF_DHNDA-BTH_/GCE (curve c) in 0.1 M KCI solution with 5.0 mM [Fe(CN)_6_]^3−/4−^. As shown in Figure 3a, a pair of redox peaks of [Fe(CN)_6_]^3−/4−^ with superior reversibility appeared on the bare GCE. After the COF_DHNDA-BTH_ was modified on the GCE, the peak current of [Fe(CN)_6_]^3−/4−^ significantly decreased, while the peak-to-peak potential difference increased. This might have occurred because the COF_DHNDA-BTH_ material hindered the transmission of electrons. After the AChE was further loaded, the peak current of the [Fe(CN)_6_]^3−/4−^ further decreased, and the peak-to-peak potential difference became wider, because the electrical conductivity of the modified electrode was further reduced by the enzyme loading, which also indirectly proved the successful fixation of AChE on COF_DHNDA-BTH_/GCE. The atom force microscopy image also proved that the enzyme was modified on the COF (Figure S6, Supporting Information). The charge transfer resistance (*R*ct) values of bare GCE, COF_DHNDA-BTH_/GCE, and AChE/ COF_DHNDA-BTH_/GCE were 40, 1293, and 2744 Ω, respectively (the error values of the resistance values were 6.22%, 3.9%, and 6.56%, respectively) (Figure 3b). The result was consistent with that of CVs, further indicating the successful preparation of the AChE/COF_DHNDA-BTH_/GCE.

### 3.4. Electrochemical Detection of Carbaryl Based on AChE/COF_DHNDA-BTH_/GCE

**Figure 4:**
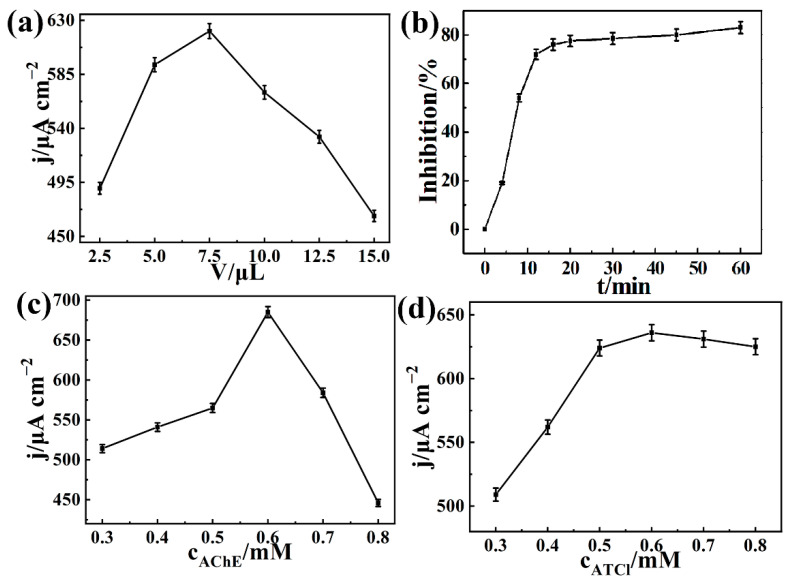
(**a**) Effect of COF_DHNDA-BTH_ dosage on AChE/COF_DHNDA-BTH_/GCE on peak current density of 5.0 mM [Fe(CN)_6_]^3−/4−^. (**b**) Plot of inhibition rate versus incubation time. (**c**) Plot of peak current density versus AChE concentration. (**d**) Relationship between concentration of ATCl and peak current density in 0.1 M KCl with 5.0 mM [Fe(CN)_6_]^3−/4−^.

Firstly, the loading amount of COF_DHNDA-BTH_ on the AChE/COF_DHNDA-BTH_/GCE was optimized by dropping different volumes of 2 mg/mL COF_DHNDA-BTH_ onto the GCE. As shown in Figure 4a, with the amount of COF_DHNDA-BTH_ dropped onto the COF_DHNDA-BTH_ increased, its peak current also increased, because the more COF_DHNDA-BTH_ was present, the more active loading sites were used to bind AChE. The maximum volume value was 7.5 μL. When the amount of COF_DHNDA-BTH_ further increased, the peak current gradually decreased because too much COF_DHNDA-BTH_ was loaded on the electrode, which led to an accumulation on the electrode surface, which hindered the conductivity of the electrode. As shown in Figure 4b, with the increase in soaking time, the inhibition rate gradually increased and the inhibition rate reached the maximum value after 12 min, and then remained stable. Therefore, 12 min was selected as the final incubation time. In the experiment, the dosage of AChE and ATCl and their reaction times affected the detection performance. Therefore, the loaded amount of AChE on COF_DHNDA-BTH_/GCE was also optimized, as shown in Figure 4c. When the amount of AChE was less than 0.6 mM, the corresponding peak current density increased with the increase in AChE. The amount of AChE was greater than 0.6 mM, and the peak current density gradually decreased because excess AChE accumulated on the COF_DHNDA-BTH_/GCE surface to block electron transport. Therefore, 0.6 mM AChE was selected as the final drop amount. As shown in Figure 4d, the content of TCl was related to the amount of ATCl and, accordingly, the amount of ATCl also needs to be optimized. When the amount of ATCl was less than 0.6 mM, the current density increased with the increase in ATCl. When the amount of ATCl exceeded 0.6 mM, the current density slightly decreased. Therefore, 0.6 mM ATCl was selected as the final detection concentration.

**Figure 5:**
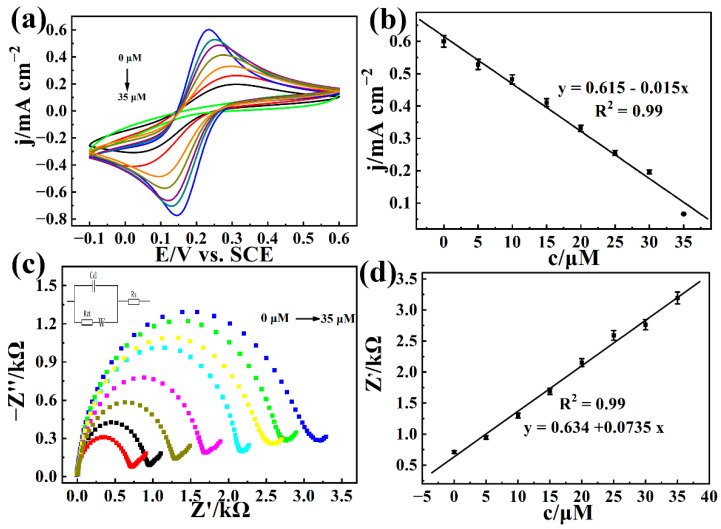
(**a**) CVs and (**c**) EIS (inset is its equivalent circuit) of AChE/COF_DHNDA-BTH_/GCE in 0.1 M KCl + 5.0 mM [Fe(CN)6]^3−/4−^ with different concentrations of carbaryl. (**b**,**d**) Corresponding linear relationship curves.

Under optimized experimental conditions, cyclic voltammetry was used to study the detection effect of AChE/COF_DHNDA-BTH_/GCE in a 0.1 M KCl solution containing 5.0 mM [Fe(CN)_6_]^3−/4−^ for different concentrations of carbaryl. As shown in Figure 5a, the peak current density gradually decreased with the increase in carbaryl concentration. This occurred because the higher the concentration of carbaryl, the more obvious the inhibition effect on AChE activity, resulting in a decrease in the amount of TCl produced; thus, the content of [Fe(CN)_6_]^3−/4−^ attracted on the electrode surface also decreased. Figure 5b shows the linear relationship between the peak current density and the carbaryl concentration. The detection limit of the biosensor was 0.23 μM (S/N = 3), and the linear range was 0.69–35 μM. As shown in Figure 5c, with the increase in carbaryl concentration, due to the inhibiting effect of carbaryl on AChE, less TCl was produced, and the corresponding impedance value was larger. Figure 5d shows the linear fitting diagram of the corresponding impedance value and carbaryl concentration. The corresponding detection limit was 0.13 μM (S/N = 3), and the linear range was 0.39–35 μM. Finally, we compared the performance of our biosensor with that of other biosensors (Table 1), and the results showed that our biosensor had a wider linear range and better sensitivity. Its good performance might be ascribed to the good catalytic activity of AChE in the constrained environment of COF_DHNDA-BTH_’s pore.

**Figure 6:**
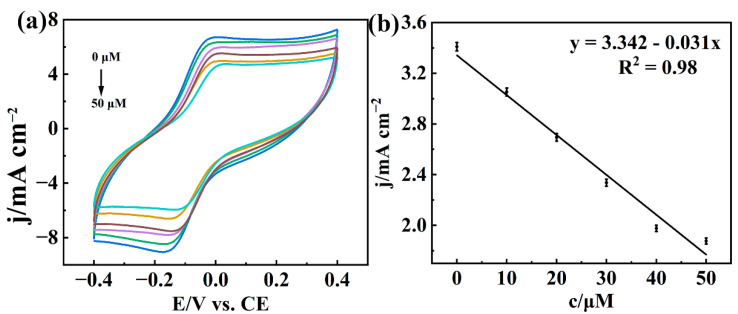
(**a**) CVs of AChE/COF_DHNDA-BTH_/portable paper-based electrode in 0.1 M KCl + 5.0 mM [Fe(CN)6]^3−/4−^ with carbaryl different concentrations. (**b**) Corresponding linear relationship curve.

Finally, the current response of the AChE/COF_DHNDA-BTH_/portable paper-based electrode was measured with the accumulation of carbaryl concentration. Figure 6a shows the results of the peak current response increase with the increase in carbaryl concentration, and the linear range was 0.48~35 μM. Figure 6b shows the corresponding linear relationship between peak current density and carbaryl concentration. The linear equation was j_p_ = –0.031c +3.342 (R^2^ = 0.98), where j_p_ and c are peak current density and carbaryl concentration, respectively. The detection limit of the paper-based electrochemical biosensor was 0.16 μM (S/N = 3). The results showed that the AChE/COF_DHNDA-BTH_/portable paper-based electrode biosensor has good performance. In general, compared with the three-electrode system composed of glassy carbon electrodes, the experimental results of paper-based electrodes were similar, which proves that the preparation of paper-based electrodes was successful. 

### 3.5. Selectivity, Stability, and Repeatability of Electrochemical Carbaryl Biosensors

**Figure 7:**
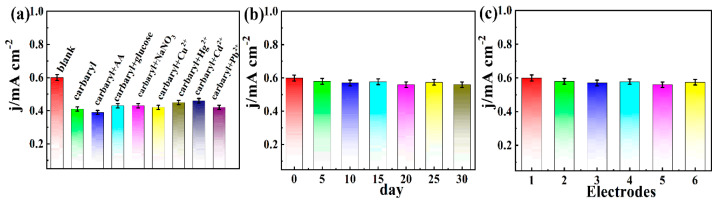
(**a**) Selectivity test of electrochemical carbaryl biosensor. (**b**) Current response histogram of electrochemical carbaryl biosensor measured for 30 consecutive days of carbaryl. (**c**) Current response histogram of six AChE/COF_DHNDA-BTH_/GCE to detect carbaryl.

As shown in Figure 7a, the selectivity of the AChE/COF_DHNDA-BTH_/GCE biosensor was studied with an interfering substance concentration five times the concentration of carbaryl. It was observed that the interfering substances including AA, glucose, NaNO_3_, Cu^2+^, Hg^2+^, Cd^2+^, and Pb^2+^ did not produce obvious interference. The good selectivity of the AChE/COF_DHNDA-BTH_/GCE biosensor could be ascribed to the low oxidation peak potential of [Fe(CN)6]^3−/4−^. Although the oxidation potential of AA was lower (about 0.15 V versus that of a saturated calomel electrode), the oxidation peak of AA was obviously different from that of [Fe(CN)6]^3−/4−^ at 0.25 V. Therefore, the AA showed obvious interference for the detection of carbaryl. The electrochemical carbaryl biosensor was stored in a refrigerator at 4 °C, and the peak current change (Figure 7b) at 0, 5, 10, 15, 20, 25, and 30 days was tested. It was observed that the peak current still maintained 93.3% of the initial value after 30 days. Next, six AChE/COF_DHNDA-BTH_/GCE were prepared for the detection of 5 μM carbaryl (Figure 7c), and the relative standard deviations (RSD) of the final result was only 3.4%. In conclusion, the electrochemical carbaryl biosensor has good selectivity, repeatability, and stability, and has a good application prospects for the detection of carbaryl. 

### 3.6. Actual Sample Testing

The content of carbaryl in lettuce was determined through a spiked experiment. The specific procedures were as follows: We chopped 100 g of lettuce (lettuce was purchased at Changsheng Market in Nanchang, Jiangxi province, on 21 September 2021). We obtained 50 μL of lettuce juice by flittering, which we then put into 5 mL of 0.1 mM KCl solution containing 5.0 mM [Fe(CN)_6_]^3−/4−^. Then, different concentrations of carbaryl standard solution were added to the above actual samples, and the concentration of carbaryl was determined by the electrochemical biosensor. The obtained recovery rates and relative standard deviation (RSD) are shown in Table 2. It was found that the spiked recoveries of three different concentrations of carbaryl ranged from 94.4% to 106% with an RSD of less than 10%. These results indicated that the electrochemical carbaryl biosensor has the potential to detect pesticide residues in real samples.

## 4. Conclusions

A new COF_DHNDA-BTH_ with abundant NH, OH, and C=O groups was synthesized. The COF_DHNDA-BTH_ presented a nanoflower-like morphology, which was composited of many 2D nanoribbons. The COF_DHNDA-BTH_ had high porosity, excellent flexibility, and good biocompatibility. Furthermore, a portable paper-based electrode with a surface of graphene-like foam was developed. The COF_DHNDA-BTH_ could be firmly assembled on the portable paper-based electrode to load AchE, whose bioactivity was greatly enhanced in the confined COF_DHNDA-BTH_ hole. The COF_DHNDA-BTH_ could better capture TCl through hydrogen bonding. The positively charged TCl attracted the anion probe [Fe(CN)_6_]^3−/4−^, which could detect pesticides at low potential (0.25 V). A turn-off electrochemical biosensor was constructed using the oxidation peak of the anionic probe [Fe(CN)_6_]^3−/4−^ as the detection signal. The linear range of the *AChE/COF_DHNDA-BTH_*/portable paper-based electrode was 0.48~35 μM, and the detection limit was 0.16 μM (S/N = 3). The paper-based electrode has broad application prospects.

## Figures and Tables

**Table 1:** The comparison of AChE/COF_DHNDA-BTH_/GCE biosensor with other biosensors.

Electrode	LOD(μM)	Linear Range(μM)	Reference
Au/PAMAM/GLUT/AChE	0.032	1.0–9.0	[50]
Carb^e^/CoPc^f^/AChE	2	5.0–75.0	[51]
Au/MPA/AChE	0.035	2.0–30.0	[52]
GC/rGO/AChE	0.0019	0.2–10	[53]
GC/PANI/MWCNT/AChE	1.4	9.9–49.6	[54]
AChE/PDDA-MWCNTs-GR/GCE	0.001	0.255–14.9	[55]
GC/MWCNT/CoPc	0.0055	0.33–6.61	[56]
AChE–MWCNTs/GONRs/GCE	0.0017	0.005–5.0	[57]
AChE/PDA-Gr/PPNWs/IdlE	0.04	0.25–7.45	[58]
AChE/COF_DHNDA-BTH_/GCE	0.23 0.13	0.69–35 0.39–35	This work

**Table 2:** Detection of carbaryl in lettuce juice by electrochemical carbaryl biosensor.

Sample	Added (μM)	Founded (μM)	Average Value (μM)	Recovery (%)	RSD (%, n = 3)
1	0	–		–	–
2	5	4.51, 5.10, 4.56	4.72	94.4	6.93
3	10	10.6, 10.4, 10.8	10.6	106	1.89
4	20	20.4, 19.8, 20.6	20.3	101.3	2.07

## Data Availability

The data is available under the request to the correspondence.

## Supplementary Materials

The following supporting information can be downloaded at: https://www.mdpi.com/article/10.3390/bios12100899/s1. Figure S1: Image of experimental and simulated XRD pattern for AA stacking and AB stacking structure of COF_DHNDA-BTH_. Figure S2: Image of pore size distribution of COF_DHNDA-BTH_. Figure S3. Image of π-π stacking distance of the layers of COF_DHNDA-BTH_. Figure S4: Image of BET pattern of COF_DHNDA-BTH_. Figure S5: Image of pore size distribution of COF_DHNDA-BTH_. Figure S6: Image of AFM of AChE/COF_DHNDA-BTH_.
