# Supplementary material for: A Novel Paper-Based Electrochemical Biosensor Based on N,O-Rich Covalent Organic Frameworks for Carbaryl Detection
Source: Biosensors (Basel). 2022 Oct 20;12(10):899. doi: 10.3390/bios12100899 (PMC9599374; doi:10.3390/bios12100899)
Supplement: Supplementary file 1 [file biosensors-12-00899-s001.zip › biosensors-1936136-supplementary.pdf]

Supporting Information

A novel paper-based electrochemical biosensor based on N,O-rich covalent organic frameworks for carbaryl detection

National Engineering Research Center for Carbohydrate Synthesis/Key Lab of Fluorine and Silicon for Energy Materials and Chemistry of Ministry of Education, College of Chemistry and Chemical Engineering, Jiangxi Normal University, 99 Ziyang Road, Nanchang 330022, China.

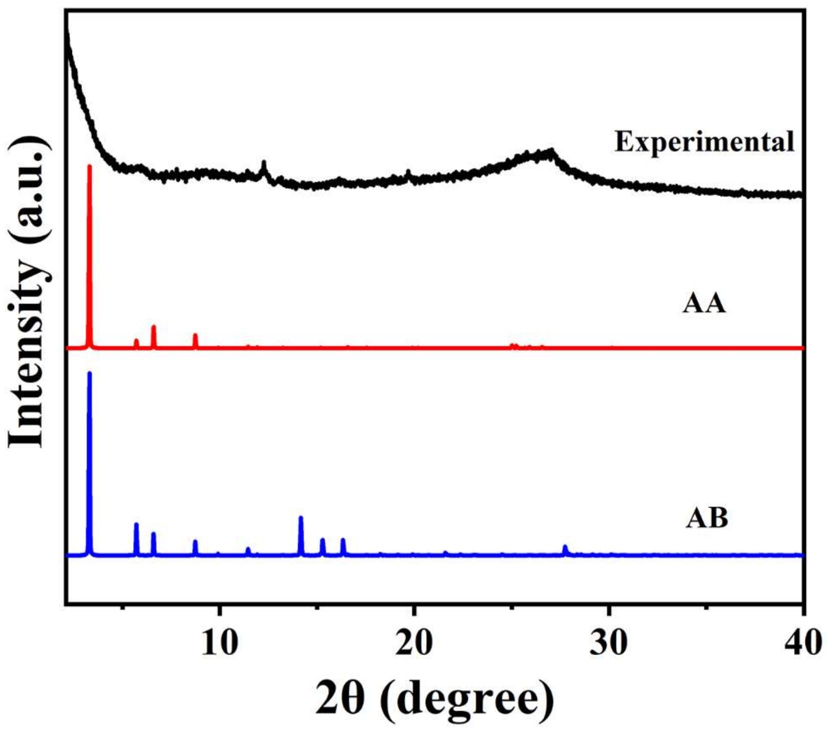

Figure S1. Image of experimental and simulated XRD pattern for AA stacking and AB stacking structure of $COF_{DHNDA\text{-}BTH}$.

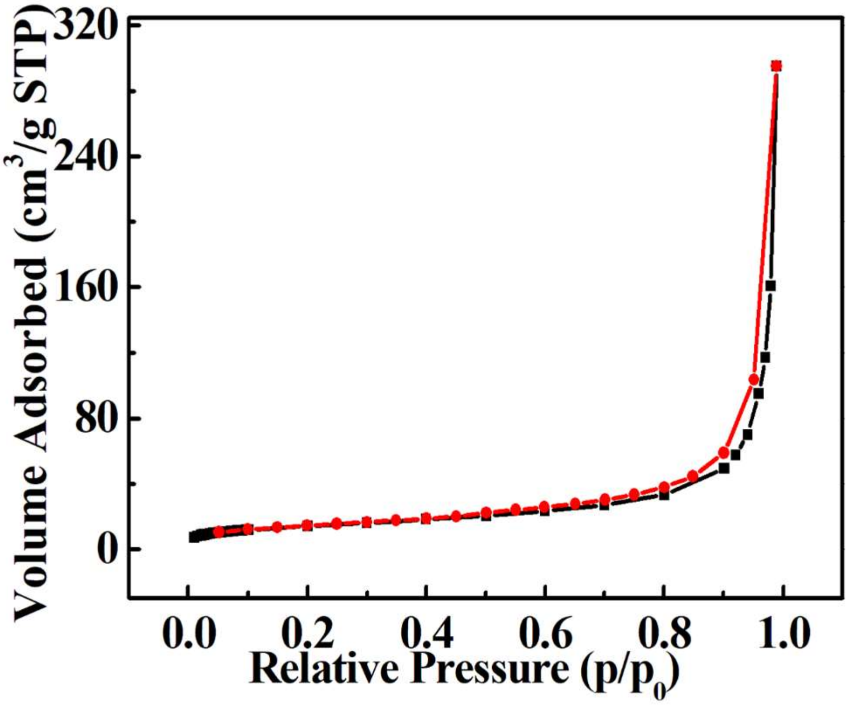

Figure S2. Image of pore size distribution of $COF_{DHNDA\text{-}BTH}$.

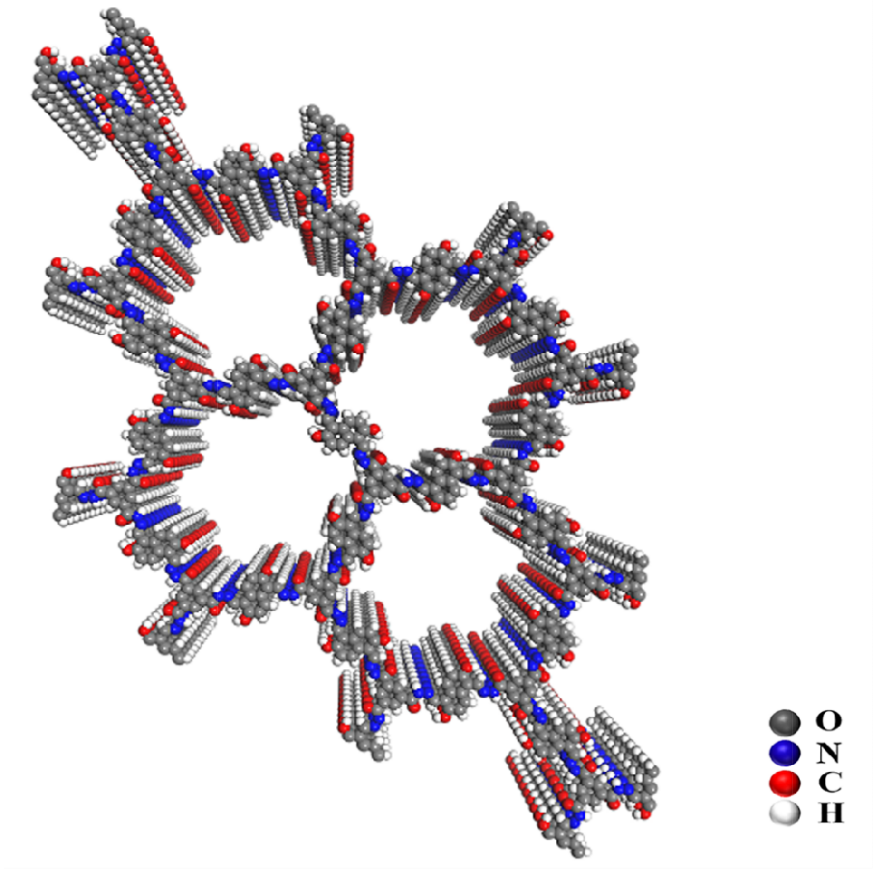

Figure S3. Image of π-π stacking distance of the layers of $COF_{DHNDA\text{-}BTH}$.

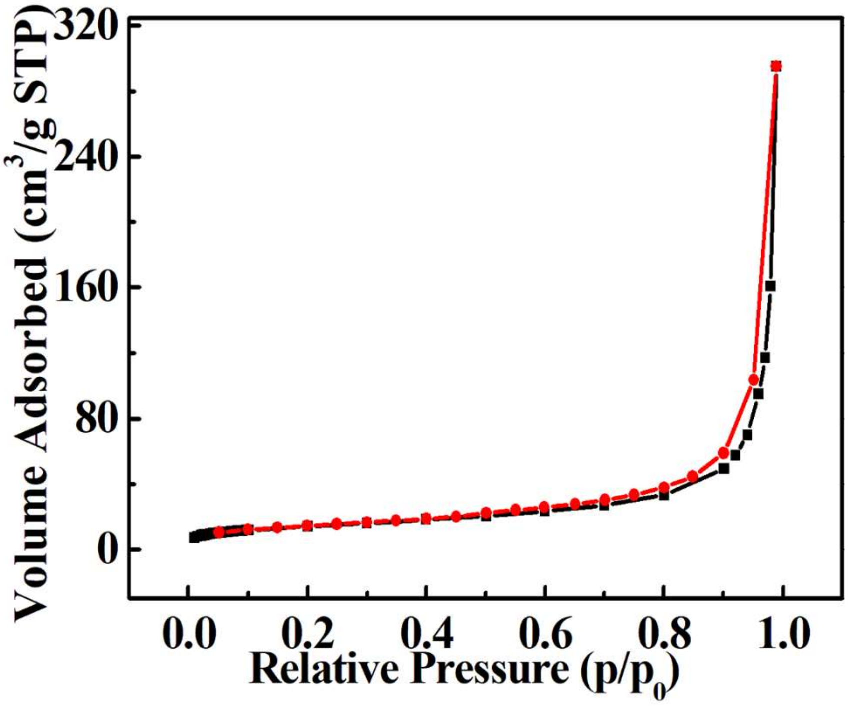

Figure S4. Image of BET pattern of $COF_{DHNDA\text{-}BTH}$.

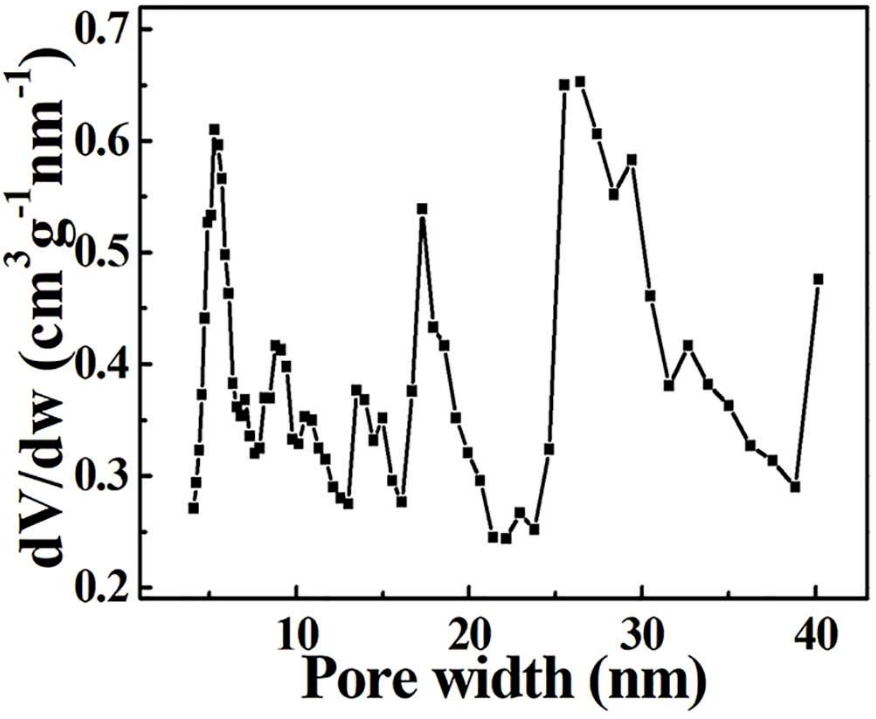

Figure S5. Image of pore size distribution of $COF_{DHNDA-BTH}$.

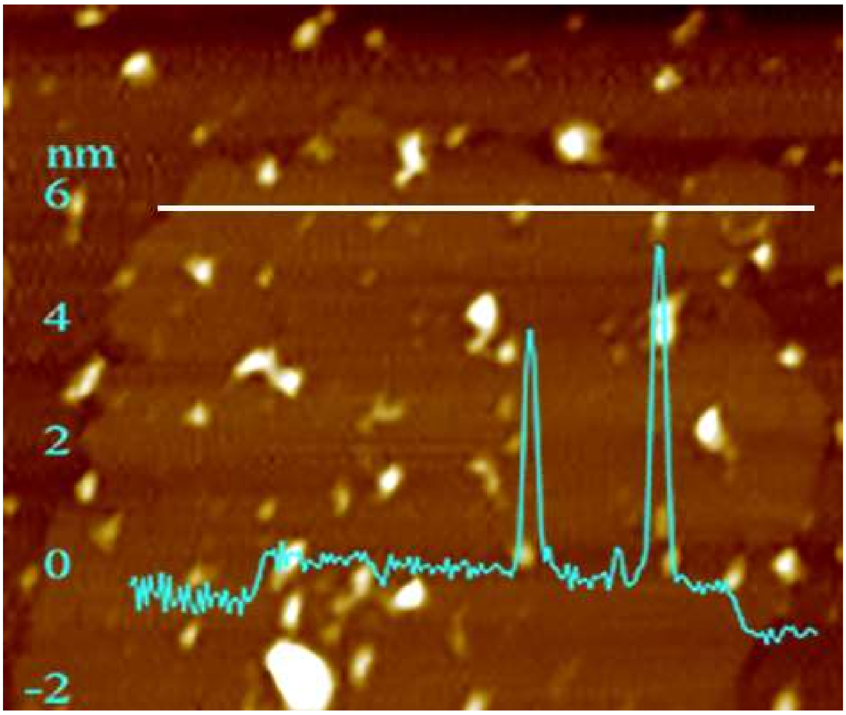

Figure S6. Image of AFM of AChE/$COF_{DHNDA\text{-}BTH}$.
